# Bisphenol A Affects on the Functional Properties and Proteome of Testicular Germ Cells and Spermatogonial Stem Cells *in vitro* Culture Model

**DOI:** 10.1038/s41598-017-12195-9

**Published:** 2017-09-19

**Authors:** Polash Chandra Karmakar, Hyun-Gu Kang, Yong-Hee Kim, Sang-Eun Jung, Md. Saidur Rahman, Hee-Seok Lee, Young-Hyun Kim, Myung-Geol Pang, Buom-Yong Ryu

**Affiliations:** 1 Department of Animal Science & Technology, Chung-Ang University, Anseong, Gyeonggi-do Republic of Korea; 2 Food Safety Risk Assessment Division, National Institute of Food & Drug Safety Evaluation, Ministry of Food and Drug Safety, Cheongju, Chungcheongbuk-do Republic of Korea; 3 National Primate Research Center, Korea Research Institute of Bioscience and Biotechnology (KRIBB), Cheongju, Chungcheongbuk-do Republic of Korea; 4 Department of Functional Genomics, KRIBB School of Bioscience, Korea University of Science and Technology (UST), Daejeon, Chungcheongnam-do Republic of Korea

## Abstract

The endocrine disruptor bisphenol A (BPA) is well known for its adverse effect on male fertility. Growing evidence suggests that BPA may interact with testicular germ cells and cause infertility as a result of its estrogenic activity. Objective of current *in vitro* study was to investigate the proliferation, survivability and stemness properties of mouse testicular germ cells exposed to BPA, and to evaluate possible expression of cellular proteome. Our results showed that germ cell viability and proliferation were not affected by low concentrations (0.01, 0.1, 1, and 10 µM) although significant reduction observed at 100 µM BPA. Germ cell self-renewal and differentiation related marker proteins expression found unchanged at those concentrations. When BPA-exposed germ cells were transplanted into recipient testes, we observed fewer colonies at higher concentrations (10 and 100 µM). Additionally, a significant frequency of recombination failure during meiosis was observed in 10 µM BPA-exposed germ cell transplanted recipient. Moreover, experiment on continuous BPA-exposed and 100 µM BPA-recovered germ cells suggested that spermatogonial stem cells are more potential to survive in adverse environment. Finally, scrutinizing differentially expressed cellular proteins resulted from our proteomic analysis, we conclude that BPA exposure might be associated with several health risks and infertility.

## Introduction

Endocrine disrupting chemicals (EDCs) are commonly known as a wide variety of substances that have the capacity of hormonal mimicry in humans and animals of all age groups. Among the EDCs produced worldwide, bisphenol A [2,2-bis(4-hydroxyphenyl)propane] (BPA) covers a large volume, as this synthetic organic compound is employed to make certain plastics and epoxy resins in several consumer products^1,2^. This chemically stable compound has estrogenic and/or anti-androgenic properties and can leach into food and water, both under normal condition and at elevated temperature^3,4^, and can consequently be accumulated in animal body^5,6^. Therefore, BPA has been a topic of debate since the discovery of its reproductive toxicity^7^, health risks even at low doses^8^, and ability to enter in various endocrine related pathways^9^. Previous studies have shown that BPA, at both high and low concentrations, has marked effects on growth, maintenance and apoptosis related signaling in various cell types, including male germ cells^10–13^. Moreover, BPA has been shown to have vertically transferred effects on spermatozoa of F1 mice following exposure in gestational period^14^, and effects on spermatozoa *in vitro* ^15^.

Spermatogenesis is a process in which tissue-specific stem cells, spermatogonial stem cells (SSCs), proliferate and grow through several rounds of mitosis and meiosis processes, and SSCs maintain a specialized balance of different cell pools through self-renewal, differentiation, and controlled apoptosis which is essential for the maintenance of male fertility^16,17^. Several endocrine and paracrine signals are involved in this complex process, coordinating SSC to undergo mitotic cell division for self-renewal and differentiation of descendant cells, along with meiotic division to generate spermatozoa^16^. It is needless to say that hormones need to bind with their cell- or tissue-specific receptors to complete cell signaling and activation. As an EDC, BPA has the potential to work as a weak estrogen receptor α/β (ERα/β) agonist^18^ and to alter hormonal signal through binding to ERs on male germ cells. So, BPA related devastating effects such as low spermatozoa count^19^, sperm protein alteration^20^, toxicity development in spermatozoa via DNA damage^21^, DNA methylation^22^ and oxidative stress^23^ have been studied well. Moreover, studies like BPA induced disruption in meiotic progression during spermatogenesis^24^ and reduction in chromosome crossover^25^ have also been reported. Therefore, these findings indicate the necessity of investigating the effects of BPA exposure on spermatogonia because spermatogenesis and spermiogenesis commence with the differentiation of SSCs. Additionally, expression of marker proteins that indicate the active physiological state of undifferentiated and differentiated spermatogonia have been specified. Among these proteins, promyelocytic leukaemia zinc finger (PLZF) has been reported to be essential for regulating self-renewal of SSCs, that is, to maintain SSCs in an undifferentiated state and maintain the SSC pool^26^. Similarly, undifferentiated SSC specific surface receptors such as glial cell line-derived neurotrophic factor family receptor alpha 1 (GFRα1)^16^ and receptor tyrosine kinase (RET)^27^ have been identified, whereas tyrosine-protein kinase or C-KIT, a marker of differentiated spermatogonia, has been described as a negative marker of SSCs at the self-renewal stage^28^. Thus, it is important to analyze the effect of BPA on the expression of these marker proteins. Several experiments have revealed that BPA has remarkable effects on the *in vitro* proliferation of germ cells and Sertoli cells at environmentally relevant concentrations, even at nanomolar levels^29,30^. However, the precise molecular mechanisms underlying how BPA affects on the stemness properties and development of spermatogonia are poorly understood. Therefore, it is necessary to determine the level of BPA effects on the inhibition or up-regulation of germ cell proliferation, expression of spermatogonia related marker proteins, germ cell stemness properties and differential expression of cellular proteins along with germ cell sustainability under long-term BPA administration.

Based on the previous findings related to the *in vivo* effects of BPA on testicular germ cells, we conducted this study to observe proliferation, growth, survivability, and apoptotic rate of these specialized cells cultured with different BPA concentrations and to examine the differential expression of germ cell markers in these cultured cells. Additionally, we tried to find out the capacity of SSCs to retain stemness properties with the investigation of meiotic abnormalities at different stages of spermatogenesis. We also conducted prolonged BPA exposure to germ cells *in vitro* to observe effects on survivability and stemness properties. Furthermore, BPA induced alteration in the expressions of cellular proteins were studied using proteomic analysis tools.

## Results

### BPA hinders testicular germ cell proliferation

**Figure 1:**
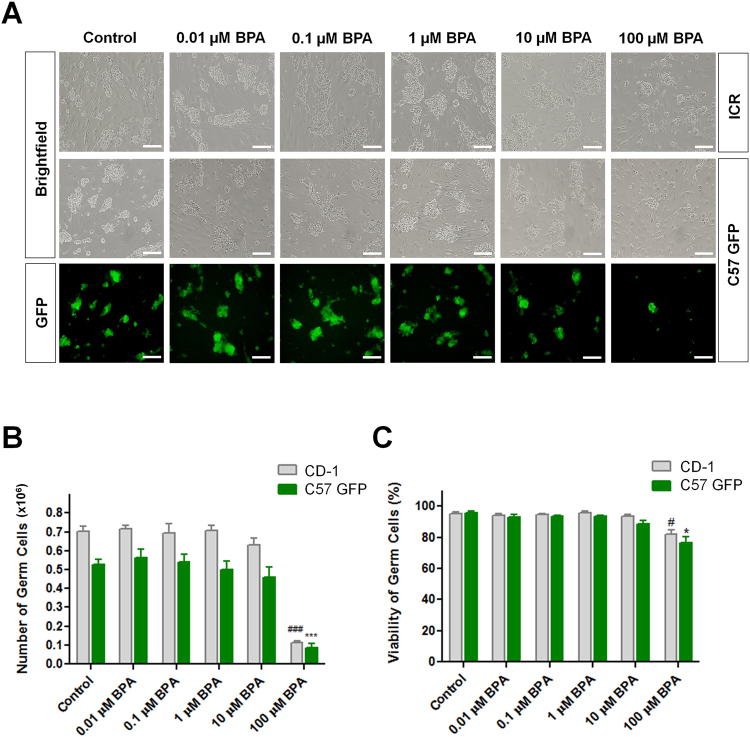
Effects of bisphenol A (BPA) on testicular germ cell proliferation. Microscopic view of *in vitro* proliferated germ cells enriched for spermatogonial stem cells with different concentrations of BPA, (**A**) brightfield image of CD-1 cell line (upper panel), C57 GFP cell line (middle panel) and fluorescent image of C57 GFP cell line (lower panel) (Scale bars = 200 μm). (**B**) Total number of proliferated germ cells and (**C**) the percentage of viable cells. Data are presented as means ± SEM of 4 independent experiments. Values with different superscript characters (#, *) indicate significant difference among the cell types (CD-1 and C57GFP) and are analyzed by one-way ANOVA (^#,^*compared with control, P < 0.05; ^###,^ ***compared with control, P < 0.001).

Firstly, we used germ cell lines from ICR (CD-1) and C57 GFP transgenic mice for the visual comparison of cultured cells under brightfield and fluorescent microscope (Fig. 1A). BPA was administrated to CD-1 and C57 GFP germ cell lines ranging from 0.01 to 100 µM in a 10-fold increasing pattern and cells were cultured for 1 week to examine cell proliferation and viability. There was a sharp decline in germ cell number (Fig. 1B) and remarkable decrease in viability at highest BPA concentration (100 µM) following the decline starting point at 1 µM BPA (Fig. 1C). We observed similar patterns of cell proliferation and viability for both wild-type (CD-1) and transgenic (C57 GFP) mice. So, we planned to use transgenic cell line for the subsequent experiments as it is easily visualized in recipient testis after germ cell transplantation. For every set of BPA-treated cultures, we also prepared control cultures where cell count and viability were optimum which indicated the utmost culture conditions.

### BPA induces apoptosis of cultured SSCs

**Figure 2:**
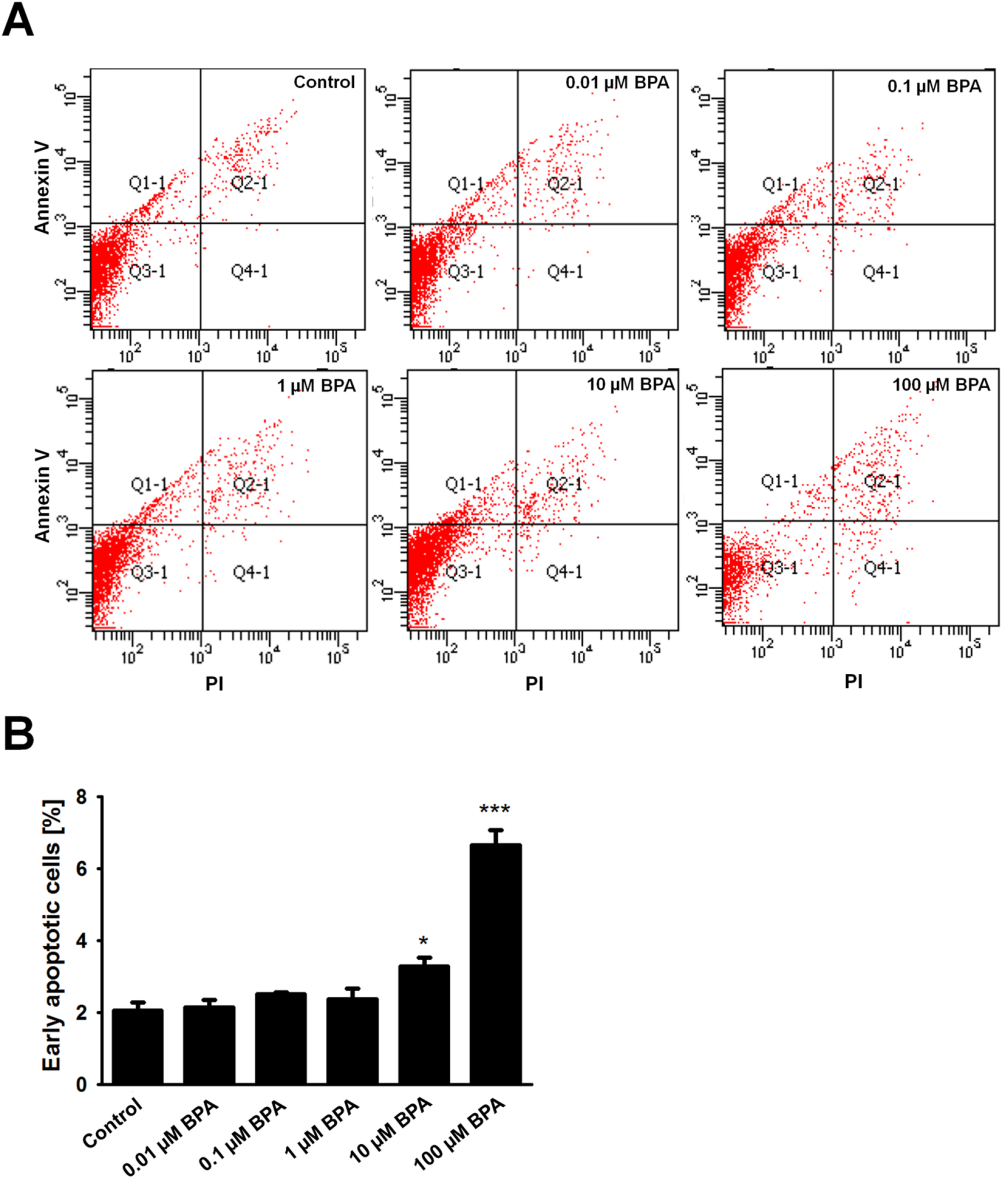
Bisphenol A (BPA)-induced apoptosis of germ cells enriched for spermatogonial stem cells. (**A**) Flow cytometric determination of apoptotic rate of BPA-treated cultured germ cells, stained with Annexin V/PI. (**B**) The percentage of early apoptotic cells is presented in a bar graph (n = 6). *Compared with control, P < 0.05; ***compared with control, P < 0.001.

We observed that the highest concentration of BPA caused damage to germ cell viability and proliferation. Therefore, we intended to observe the percentage of apoptotic cells among the treatment groups. Harvested germ cells were used immediately for staining of Annexin V and propidium iodide (PI) and we detected significantly higher percentages of apoptotic cells in 10 µM BPA and 100 µM BPA treatment groups. 0.01, 0.1, and 1 µM BPA-exposed cells showed that the percentage of apoptotic cells were similar to control. Highest proportion of apoptotic germ cells were found in 100 µM BPA treatment (Fig. 2A and B).

### Evaluation of spermatogonia related marker proteins

**Figure 3:**
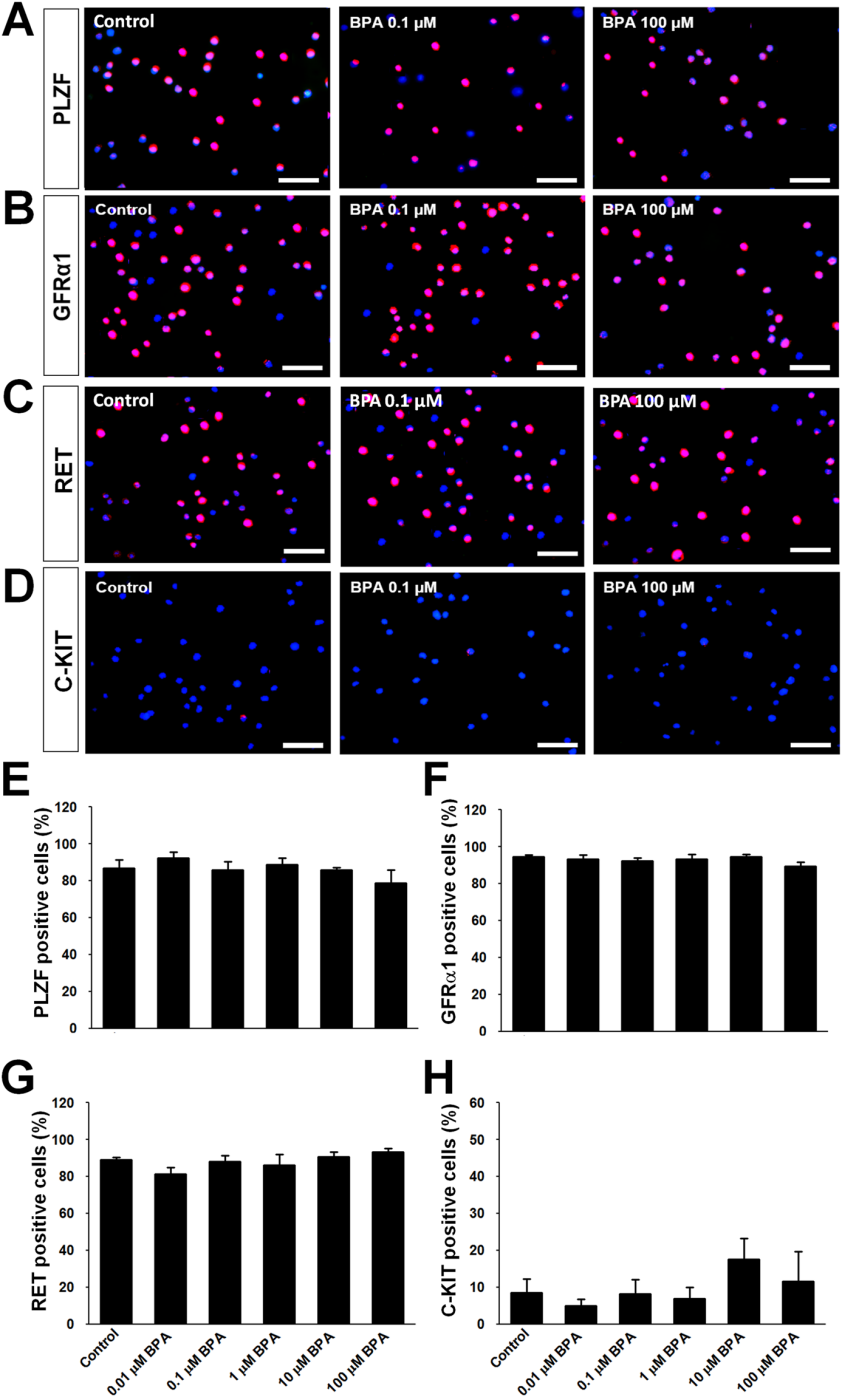
Spermatogonia related marker proteins’ expression in bisphenol A (BPA)-treated cultured germ cells enriched for spermatogonial stem cells. Immunostaining of undifferentiated spermatogonia markers’ expression in 1 week BPA-administrated cultured germ cells are indicated as red fluorescence, (**A**) PLZF, (**B**) GFRα1 and (**C**) RET. Pictures of low BPA treatment (0.1 µM BPA) and high BPA treatment (100 µM BPA) are given because the expression patterns of all treatment groups are visually similar. (**E, F,** and **G**) The percentage of these markers’ expression among total cells showed sequentially as bar graph. (**D**) The expression of differentiated spermatogonia specific marker C-KIT in 1 week BPA-administrated cultured germ cells (red). (**H**) Bar graph presentation of the percentage of C-KIT positive cells. Data were obtained from 6 independently established cultures with different BPA treatments. Cells were counterstained with DAPI (blue). Scale bars = 50 μm.

To confirm undifferentiated spermatogonia related marker proteins PLZF, GFRα1 and RET, we investigated their expression patterns in BPA-treated cultured germ cells. In immunocytochemistry, there were no significant differences observed in the percentages of PLZF, GFRα1 and RET positive germ cells (Fig. 3A, B, C, E, F, and G). Differentiated spermatogonia related marker protein C-KIT was also examined. C-KIT was expressed in a very small percentage of cells with no significant differences among BPA treatments and control (Fig. 3D and H).

### Effects of BPA on SSCs stemness properties

**Figure 4:**
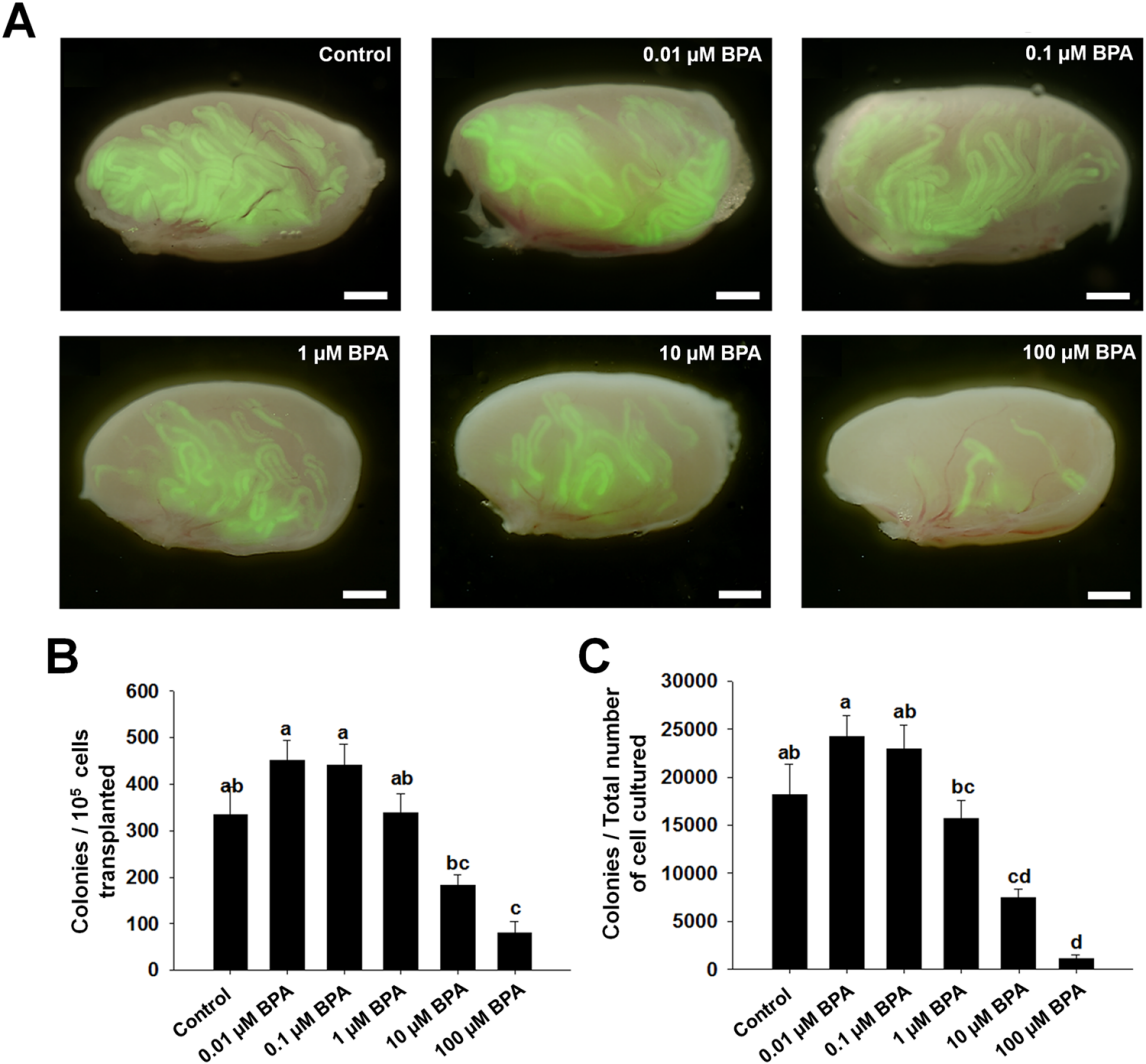
Effects of bisphenol A (BPA) treatment on spermatogonial stem cell (SSC) activity. (**A**) Testes of recipient mice showing GFP expressing colonies of donor SSCs 2.5 months after transplantation of BPA treated cultured germ cells. Scale bars = 2 mm. (**B**) The number of colonies per 10^5^ cultured germ cells presented as bar graph and (**C**) the relative numbers of colonies from total number of cultured germ cells. Total numbers of mice/testes analyzed were 6/10, 7/14, 7/13, 8/15, 10/19, and 9/16 for control, 0.01, 0.1, 1, 10, and 100 µM BPA concentrations, respectively. Different letters (a, b, c, and d) indicate significant difference (P < 0.05) between treatments.

Germ cells treated with different concentrations of BPA were transplanted into recipient C57/BL6 mice. After 2.5 months, the transplanted testes were collected, and donor cell derived C57 GFP positive colonies were counted under Nikon AZ100 microscope (Fig. 4A). Quantification of donor cell derived colonies indicated that SSCs among the cultured germ cells treated with low BPA concentrations (0.01, 0.1, and 1 µM) could not alter their stemness properties. At relatively higher BPA concentrations (10 and 100 µM), SSCs could produce significantly less colonies compared to the number of transplanted germ cells and total number of cultured germ cells (Fig. 4B and C). This result indicated that stem cell like characteristics of SSCs could reduce significantly with BPA exposure.

### BPA can induce meiotic abnormalities in SSCs

**Figure 5:**
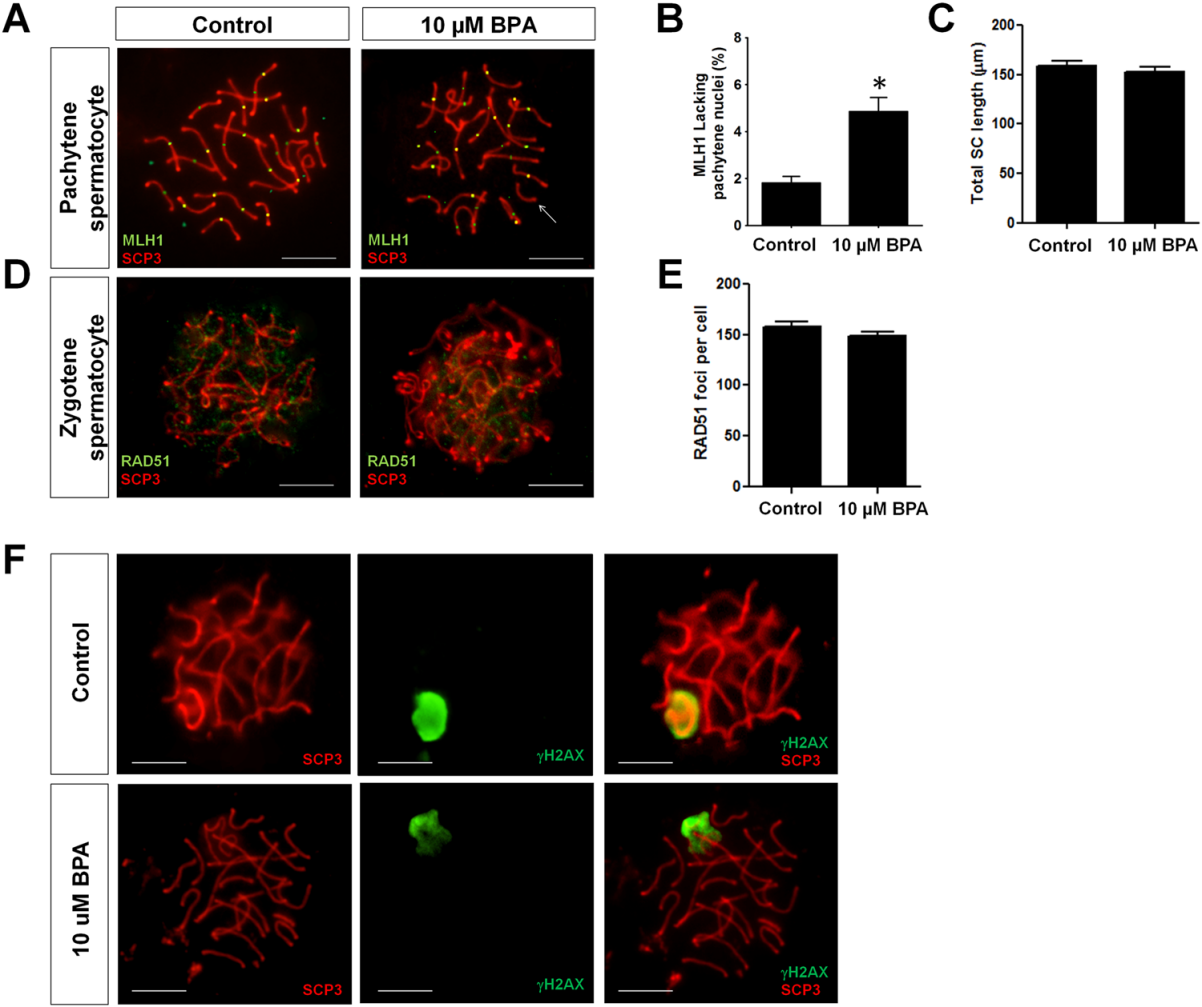
Analysis of meiotic abnormalities during spermatogenesis in recipient mice transplanted with bisphenol A (BPA)-treated cultured germ cells. (**A**) Representative pachytene spermatocytes of control and 10 µM BPA treatment group. White arrow indicates a synaptonemal complex (SC) lacking an MLH1 focus in 10 µM BPA treatment group. MLH1 = green, SYCP3 = red. (**B**) The frequency of cells with an SC lacking MLH1 in control and 10 µM BPA treatment group (*compared with control, P < 0.05). (**C**) Total autosomal SC length (μm) in pachytene cells from control and 10 µM BPA treatment group. (**D**) Representative zygotene spermatocytes from control and 10 µM BPA treatment group showing RAD51 foci. RAD51 = green, SYCP3 = red. (**E**) Bar graph represents RAD51 foci count in control and 10 µM BPA treatment group. (**F**) γH2AX foci formation in pachytene spermatocyte. For both control and BPA 10 µM BPA treatment group, γH2AX foci is localized only in sex body (X and Y chromosomal region), not in autosomes. Scale bars in all pictures = 10 µm.

At a concentration of 10 µM, BPA did not affect the viability and proliferation of germ cell *in vitro* but showed significantly higher proportion of apoptotic cells. Additionally, we observed that treatment of SSCs with 10 µM BPA resulted in fewer colonies in the recipient testes after transplantation. Therefore, we intended to examine some proteins expression pattern related to meiotic stages and that was why DMSO (control) and 10 µM BPA-treated germ cell transplanted mice were analyzed for this experiment. Among the nuclei of pachytene spermatocyte, we did not observe any difference in synaptonemal complex (SC) length of autosomes between control and 10 µM BPA treatment group (Fig. 5C); however, chromosomes that lacked MutL homolog 1 (MLH1, crossover-associated protein) foci were found significantly high in 10 µM BPA treatment group (Fig. 5A and B). This result indicates that BPA exposure can trigger germ cells to produce “crossover-less” SCs and can increase the frequency of recombination failure. We made our next move to examine the number of RAD51 (assists in repair of DNA double strand breaks) foci among zygotene spermatocyte nuclei. We did not find any remarkable difference between control and 10 µM BPA treatment group (Fig. 5D and E). Finally, we checked the expression of meiotic DNA double-strand breaks (mDSB) indicating protein γH2AX in pachytene nuclei. γH2AX is spatially and temporally linked to DSB in leptotene and zygotene spermatocytes stages, disappears from synapsed autosomes in the pachytene stage but remains on the sex chromosomes^31^. Consistent with these results, we observed expression of γH2AX only in the unpaired regions of the X and Y chromosomes (Fig. 5F).

### Effects of long term BPA exposure on germ cell enriched for SSCs

**Figure 6:**
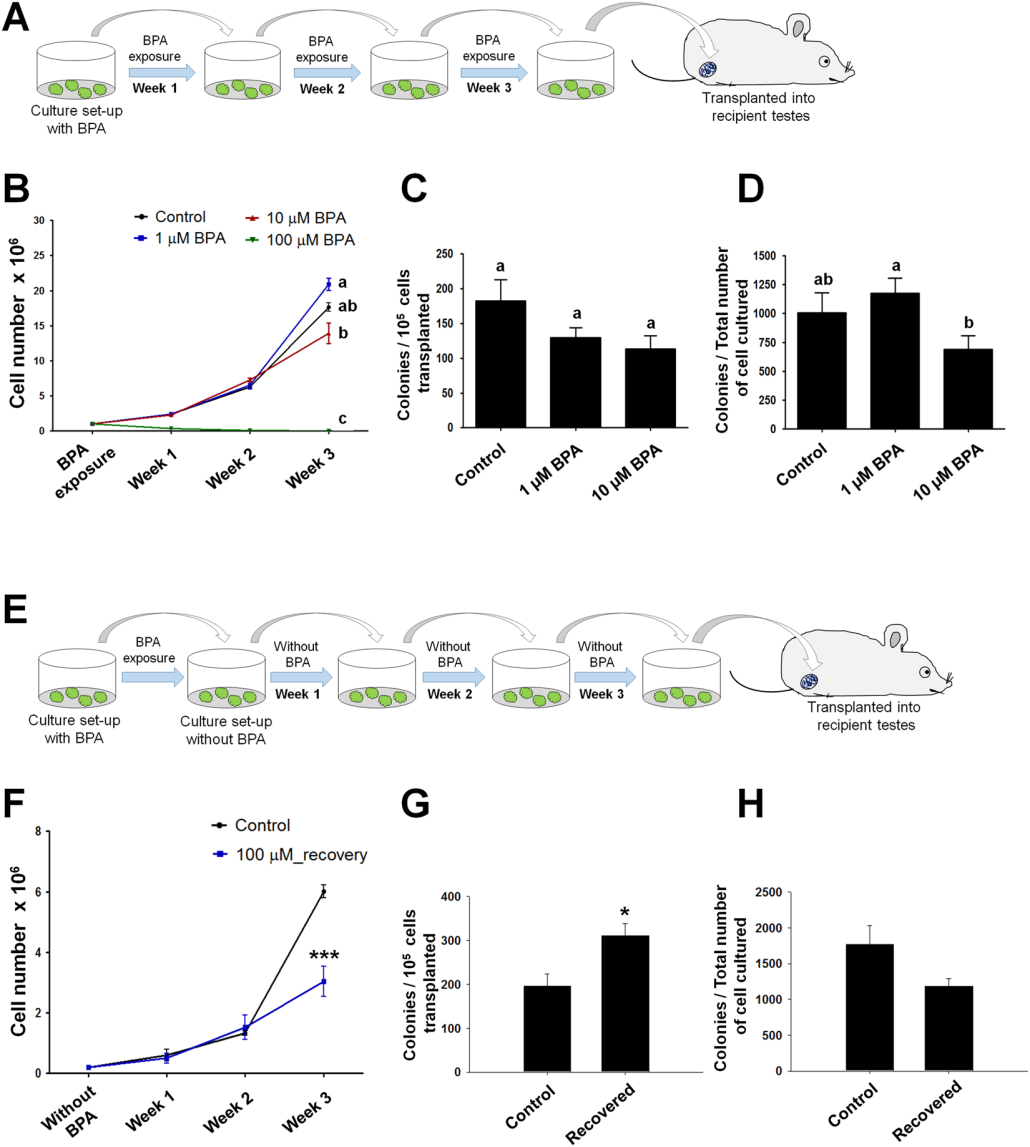
Bisphenol A (BPA) effect on stemness activity of spermatogonial stem cells after long-term BPA exposure and recovery from 100 µM BPA treatment. (**A**) Diagram of continuous BPA exposure method and subsequent transplantation into recipient mice. (**B**) Germ cell numbers following BPA exposure in culture for 3 consecutive weeks (n = 5). Significant differences (P < 0.05) analyzed by one-way ANOVA are indicated by different letters (a, b, and c). Long-term BPA-exposed germ cells transplanted into recipient mice. Ratios of the number of donor cell derived colonies (**C**) per 10^5^ transplanted germ cells and (**D**) total number of cultured germ cells are showed as bar graph. Total numbers of mice/testes analyzed were 8/14, 6/11 and 7/10 for control, 1, and 10 µM BPA concentrations, respectively. Different letters (a and b) indicate significant differences (P < 0.05) between treatments. (**E**) Diagram of the method of recovered germ cells from 1 week 100 µM BPA exposure and subsequent transplantation into recipient mice. (**F**) Germ cell numbers of control and recovered cells from 100 µM BPA exposure are cultured for 3 consecutive weeks without BPA (n = 5). Analysis of donor derived colonies of recovered germ cells (**G**) per 10^5^ transplanted germ cells and (**H**) total number of cultured germ cells. Total numbers of mice/testes analyzed were 9/16 and 6/12 for control and 100 µM BPA recovered, respectively. Values with superscript characters (*) indicate significant differences analyzed by t-test (*compared with control, P < 0.05; ***compared with control, P < 0.001).

To investigate germ cell proliferation strategy in case of continuous BPA exposure, we conducted a long term (at least for 3 weeks) exposure of different concentrations of BPA (Fig. 6A). The cultured cell proliferation rate was evaluated and we chose 1, 10, and 100 µM BPA concentrations according to their effects found at cellular level. As shown in Fig. 6B, proliferation of 1 and 10 µM BPA exposed germ cells increased gradually from week 1 to week 3 and cell numbers were found significantly different between 1 and 10 µM BPA at week 3. A dramatic decrease of cell proliferation was observed at 100 µM BPA treatment; only a small percentages of cells seeded at week 1 survived until week 3 (Fig. 6B). Therefore, to investigate the functional status of SSCs after long exposure to BPA, transplantation to recipient mice were performed with germ cells exposed to control, 1, and 10 µM BPA for 3 weeks. 100 µM BPA-treated cultured cells were excluded due to the small number of cells harvested, which was insufficient for transplantation. Colonies were counted 2.5 months after transplantation, and the results indicated that the activity of SSCs subjected to long-term BPA exposure decreased in a dose-dependent manner, but no significant difference was observed compared with control group (Fig. 6C). However, analyzing colony number among the total number of cultured germ cells, we observed a significant reduction in colony number in case of continuous exposure to 10 µM BPA compared with 1 µM BPA (Fig. 6D).

### Recovered SSCs from high BPA exposure increases colonization

A minute proportion of cells was observed to survive *in vitro* against 1 week 100 µM BPA treatment. We tried to scrutinize the proliferation and stem cell like abilities of the surviving germ cells. Therefore, 100 µM BPA was applied to germ cell culture for 1 week and then survived cells (recovered germ cells) were used to set a new BPA-free culture panel for three consecutive weeks (Fig. 6E). We did not observe any remarkable difference in the number of recovered germ cell proliferation between control group and recovered group during the first 2 weeks; however, during 3^rd^ week of culture, recovered group proliferated more slowly than control with a significant difference in cell count (Fig. 6F). We performed germ cell transplantation into recipient mice to check the stemness properties of 100 µM BPA-recovered SSCs. We counted significantly higher number of SSC colonies in case of 100 µM BPA-recovered germ cells than control (Fig. 6G) but the total number of colonies of recovered cells was not significantly different compared with control (Fig. 6H).

### 2-DE analysis of BPA-induced proteome alterations in germ cells

A two-dimentional gel electrophoresis (2-DE) based method was applied for comparative proteomic analysis and to observe the changes in protein expression patterns after BPA treatment in germ cell culture. On average, 227 protein spots were detected in each gel among which 45 different spots showed dose dependent expression profile. Significant (P < 0.05) changes were noticed in 16 spots in case of control and 0.01, 0.1, 1, 10, and 100 µM BPA-treated groups and 9 of them were characterized as important proteins for cellular functions (Table 1). Peptidylprolyl isomerase A (PPIA), which is involved in inflammation, apoptosis, and other intracellular signaling was expressed significantly high in 100 µM BPA-treated germ cells compared with control. Similarly, expression of annexin A4 (ANXA4), which is involved in exocytic and endocytic pathways, was up-regulated in 100 µM BPA-treated germ cells compared with control. The expression of oxidative stress related protein peroxiredoxin 4 (PRDX4) and antioxidant defense initiated enzyme superoxide dismutase (SOD2) were also found as significantly high in 100 µM BPA-treated cells. Additionally, expression of heat shock proteins (HSPs) [10 kDa HSP (HSPE1), 60 kDa HSP (HSPD1) and 70 kDa HSP8 (HSPA8)] differed markedly in the 100 µM BPA-treated group; HSPE1 expression was up-regulated, while HSPD1 and HSPA8 expression were down-regulated. Glycosyl transferase group 1 (GLT1D1), an enzyme capable of producing several types of polysaccharides, was also down-regulated as a result of 100 µM BPA treatment. However, significantly higher expression of cell proliferation regulator protein prohibitin (PHB) was observed in 0.01 µM BPA- treated germ cells compared with all other groups including control (Table 1).

**Table 1:** Proteins with significantly lower or higher expressions in BPA treated germ cells and control groups.

Symbol	Protein description	gi no.	Mascot score*	Relative intensity(normalized)**					
				Control	0.01 uM	0.1 uM	1 uM	10 uM	100 uM
*Apoptosis induction*									
PPIA	Peptidyl-prolylcis-trans isomerase	gi|657170146	38.4	1.00^a^	2.25 ± 0.65^a,b^	2.42 ± 048^a,b^	2.55 ± 0.73^a,b^	2.27 ± 1.00^a,b^	3.70 ± 0.76^b^
ANXA4	Annexin A4	gi|161016799	218	1.00^a^	0.91 ± 0.34^a^	1.10 ± 0.48^a^	1.62 ± 0.31^a,b^	1.49 ± 0.52^a,b^	2.07 ± 0.09^b^
*Oxidative stress*									
SOD2	Superoxide dismutase (mitochondrial)	gi|3041732	48	1.00^a^	0.99 ± 0.22^a^	1.15 ± 0.34^a^	1.27 ± 0.28^a^	1.60 ± 0.54^a^	3.00 ± 0.12^b^
PRDX4	Peroxiredoxin 4	gi|18044557	38	1.00^a^	1.46 ± 0.25^a^	1.92 ± 0.67^a,b^	1.54 ± 0.23^a^	2.17 ± 0.96^a,b^	3.10 ± 0.66^b^
*Fertility related protein*									
PHB	Prohibin	gi|6679299	316	1.00^a^	4.18 ± 1.15^b^	1.79 ± 1.06^a,b^	2.14 ± 0.49^a,b^	2.14 ± 1.44^a,b^	2.54 ± 0.66^a,b^
*Protein conformation and prevention of unwanted protein folding*									
HSPE1	10 kDa heat shock protein (mitochondrial)	gi|6680309	96	1.00^a^	1.28 ± 0.45^a,b^	1.82 ± 0.53^a,b^	2.01 ± 0.31^a,b^	2.52 ± 0.48^a,b^	6.48 ± 1.02^b^
HSPD1	60 kDa heat shock protein (mitochondrial)	gi|183396771	195	1.00^a^	0.97 ± 0.11^a,b^	0.73 ± 0.12^a,b^	0.81 ± 0.09^a,b^	0.57 ± 0.11^a,b^	0.25 ± 0.10^b^
HSPA8	Heat shock 70 kDa protein 8	gi|51702275	64	1.00^a^	0.89 ± 0.12^a^	0.48 ± 0.10^a,b^	0.51 ± 0.07^a,b^	0.33 ± 0.09^b^	0.28 ± 0.06^b^
*Diverse range of polysaccharide formation*									
GLT1D1	Glycosyl trans- ferase group 1	gi|296924979	31.6	1.00^a^	1.23 ± 0.17^a,b^	0.85 ± 0.08^a,b^	0.79 ± 0.06^a,^	0.40 ± 0.11^b^	0.24 ± 0.08^b^

*MASCOT score is −10 log (P), where P is the probability that the observed match is a random event.

Individual scores >30 indicate identity or extensive homology (p < 0.05).

**Relative spots intensity in control and BPA-treated (μM) cultured germ cells. Data are presented as mean ± SEM (3 replicates). Values with different superscript characters (a,b,c) indicate significant differences between the control and treatment groups as determined by one-way ANOVA (P < 0.05).

**Figure 7:**
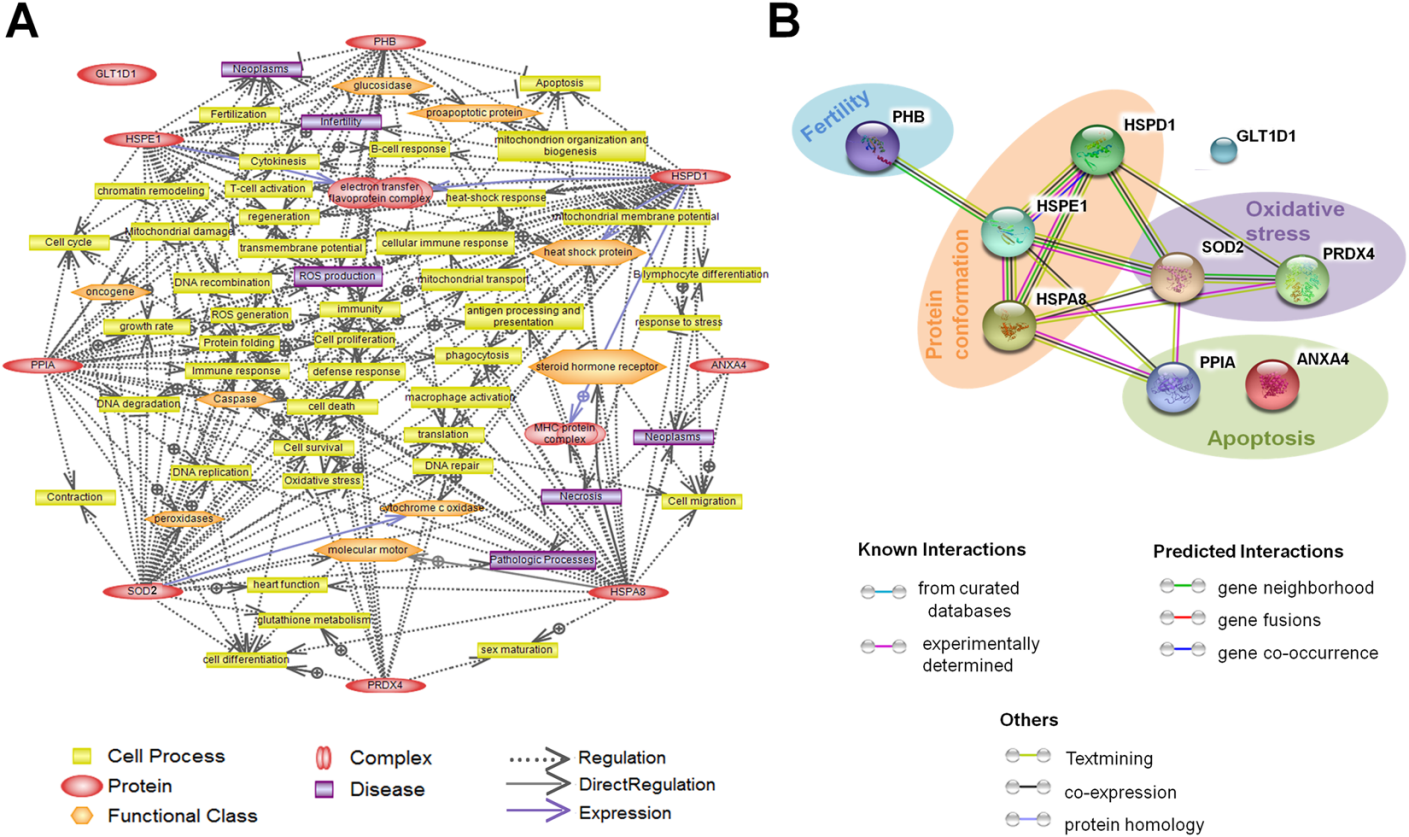
Pathways regulated by identified proteins in spermatogonial stem cells as predicted by Pathway Studio software and internal relationship of these identified proteins. (**A**) The interaction network among these detected proteins. The network showed 9 identified proteins and the Pathway Studio software was used to map them according to their protein-protein interaction. (**B**) The functional proteins association network of the identified proteins using the STRING system (online, version 10.0).

Based on the proteins detected by 2-DE, including proteins that were similarly or differentially expressed in germ cells treated with different BPA concentrations, we plotted them to observe their interrelationship. Thus, a co-relation among these proteins was observed regarding cell signaling, growth, apoptosis, and disease related pathways (Fig. 7A). Moreover, prediction of protein-protein interactions among the identified proteins based on the STRING system showed that oxidative stress and apoptosis related proteins were connected with HSPs, while HSPE1 is also associated with PHB (Fig. 7B). These protein-protein interactions were observed as gene co-occurrence, gene neighborhood and co-expression. These results suggest that even minor alterations in protein expression as a result of BPA exposure could result in up- or down-regulation of associated proteins, with wide-ranging impacts on cellular physiology.

## Discussion

The xenoestrogenic compound BPA has become an important topic nowadays because of its ability to increase the risk of several diseases and health related issues throughout 50 years. Many studies have reported that BPA has vicious effects on reproductive system and sexual behavior of both males and females, following exposure during childhood or even adulthood^32–34^. BPA can also simulate the risk of breast cancer of female if exposure occurs during perinatal or fetal development^35^, and can promote the proliferation, invasiveness and metastasis of neuroblastoma^36^. Additionally, this EDC has been discovered as one of the risk factors of thyroid disfunction^37^, asthma^38^, heart disease^39^, and obesity^40^. Therefore, researchers have conducted their experiments to characterize its involvement in the pathogenesis of these diseases along with its chemical kinetics that can disrupt or alter normal physiological and neural development, especially within the tolerance range proposed by the Food and Drug Administration (FDA).

Although the commercial use of BPA is progressively substituted by other bisphenols such as BPS (alternative in BPA-free thermal paper), BPF, and BPAF (in BPA-free plastics)^41^, some recent studies have also described them as hazardous components by characterizing their estrogenic activities^42^, negative effects on meiotic maturation of pig oocytes^43^, and above all, their presence in human plasma^41^.

BPA closely mimics the structure and function of the hormone estradiol, with the ability to bind to and activate the same estrogen receptor as the natural hormone^9^, which interferes with the function of androgens or male hormones. To clarify this, we used different concentrations of BPA in germ cell culture along with all required growth factors and maintained all necessary environmental factors. We observed devastating cell loss and a significant decline in viability at the highest BPA concentration (100 µM). High concentration of any substrates in cell culture media can hamper cell growth, and this effect can be severe for toxic compounds; thus, the profound cell loss observed in the presence of 100 µM BPA indicates that BPA is toxic to testicular germ cells at high doses.

Additionally, we observed a decline in germ cell number at micromolar (µM) concentration of BPA which suggests that this concentration level may damage the male reproductive system. Another study showed that exposure of BPA over 100 μM for 48 h was harmful to mouse Sertoli cells^30^. We found similar effect of this chemical on testicular germ cells. As BPA has estrogenic characteristic, low concentrations of BPA administration to germ cells and continuous BPA exposure in consecutive cultures were also considered as our further investigations. Considering the toxic nature of high doses (100 µM) of BPA on cell proliferation and viability, we measured the percentage of apoptotic cells in all treatment groups of cultured germ cells. We observed the highest percentage of apoptotic cells in the 100 µM BPA-treated group. However, 10 µM BPA-treated germ cells also showed a significantly higher proportion of apoptotic cells compared to control and cells treated with lower BPA doses, although significant differences in cell viability and proliferation were not observed at this concentration. These results suggest that 10 µM BPA might impair cell health and cause cell death in testicular germ cells, even though cell proliferation appeared normal in culture.

Our next move was to determine whether there was any BPA-induced change in cultured germ cell characteristics. In the process of self-renewal, cultured germ cells normally undergo numerous mitotic cell divisions, thereby increasing their number. We used specific marker proteins for self-renewal such as PLZF, GFRα1, and RET, as well as the differentiation marker protein C-KIT. Our immunocytochemistry (ICC) data indicated that there was no significant difference in the percentage of SSCs with self-renewal characteristic following treatment with various concentrations of BPA. Usually very few cultured germ cells exhibit expression of differential marker C-KIT and we observed no difference in C-KIT expression due to BPA exposure. Accumulation of these data indicate that BPA provides toxic environment to germ cell culture regarding their survivability without altering the expression of their characterized marker proteins.

Transplantation is the most accurate method available to examine the stemness properties of SSCs^44,45^ and the actual number of stem cells among cultured germ cells. Therefore, germ cell transplantation was performed in view of observing BPA induced cellular defects in SSCs. We used C57BL/6 mice in which endogenous germ cells were removed by busulfan treatment, to enable C57 GFP cells to occupy the new niche. As shown in Fig. 4B and C, a slight increase in the number of SSCs was observed at lower BPA doses (0.01 and 0.1 µM) compared to control; however, this difference was not statistically significant. This result indicates that stem cells are able to tolerate BPA at a certain concentration and continue to proliferate. Additionally, considering10^5^ transplanted germ cells and total number of cultured germ cells, dose dependent decreases in donor derived colony number were observed which indicates a decrease of functional SSCs number among BPA treated cultured germ cells, especially at higher doses. Interestingly, we observed a remarkable reduction in colony number among total number of cultured cells treated with 10 µM BPA which provided further evidence for a large population of apoptotic cells at this concentration. Therefore, based on the effects of 10 µM BPA on germ cell colonization and apoptosis, we considered this dose for further experiment related to meiotic abnormalities during spermatogenesis using spermatocytes from transplanted recipients. As shown in Fig. 5, we did not observe any change in autosomal SC length in pachytene spermatocytes (PS) between control and BPA-treated group, but did observe a significantly higher number of PS with SCs lacking at least one MLH1 focus. This could explain the result of recombination failure in unpartnered univalents at metaphase I which can promote cell death^25,46^. DNA double strand break repair protein RAD51 plays a major role in homologous recombination of DNA, we examined its expression in zygotene spermatocyte nuclei; however, no reduction in the number of RAD51 foci was observed in the BPA-treated group. Finally, we observed no expression of γH2AX in the synapsed autosomes of PS, which suggests that 10 µM BPA did not cause persistent mDSBs. Therefore, our results reveal that BPA exposure is not accompanied by a reduction in either SC length or the number of RAD51 foci, but may cause recombination failure during metaphase I of meiosis without causing any mDSB-related abnormalities.

Considering these experimental data, we decided to evaluate the proliferation and functional activities of cultured germ cells subjected to prolonged BPA exposure at three relatively high concentrations (1, 10, and 100 µM) for 3 weeks. Interestingly, 1 µM BPA-treated germ cells showed the highest proliferation at the end of week 3, suggesting that cell growth might be boosted by low concentrations of BPA^29^; however, the difference was not significantly higher than control. These findings led us to think about the stemness status of SSCs among BPA-treated germ cells. However, our transplantation data did not show any significant reduction in stemness properties or proportion of SSCs among long-term BPA treated cells. Moreover, recovered germ cells from 100 µM BPA treatment showed acceleration in cell proliferation over the weeks. It is needless to say that BPA-exposed germ cells need a period of time to be cured from the toxic stress. So, proliferation of BPA-exposed cells was remarkably lower than control even at the end of week 3. Unexpectedly, we observed a significantly higher number of donor derived colonies into recipient per 10^5^ BPA-recovered germ cells (Fig. 6G), suggesting that a higher proportion of SSCs among germ cells survived in 100 µM BPA treatment. Therefore, SSCs may be more robust to BPA administration *in vitro* among cultured germ cells and BPA-exposed SSCs can retain or regain their stemness properties during the subsequent BPA-free culture periods. However, as the total number of BPA-recovered germ cells was lower than control, we did not observe any change in colony count per total number of cultured germ cells.

Next, two dimensional gel electrophoresis (2-DE) was performed in order to determine the number and level of expressed proteins related to several cellular functions. It was observed that proteins related to apoptosis and oxidative stress, as well as heat shock proteins exhibited significantly high responses at 100 µM BPA. Expression of inflammation and apoptosis related protein PPIA, which has roles in intracellular signaling, protein folding and assembly, and cell cycle regulation^47^, was elevated in germ cells cultured with 100 µM BPA. One of the members of annexin family, ANXA4, has already been reported as subcellular localization from the nucleus to the cytoplasm in the time of apoptosis^48^. Evaluating the elevated expression of this protein, we hypothesize that higher concentration of BPA triggers apoptosis. Similarly, antioxidant defense protein PRDX4 and oxidative stress related mitochondrial enzyme SOD2 were highly expressed in germ cells treated with the highest BPA dose. PRDX4 controls cytokine-induced peroxide levels and thereby mediates signal transduction in mammalian cells. It is known that spermatogenic cells lacking PRDX4 are more susceptible to cell death via oxidative damage than their wild-type counterparts^49^, and that PRDX4 exerts its protective function against oxidative damage by scavenging reactive oxygen species (ROS) in the extracellular space^50^. On the other hand, studies have proven that SOD2 is a biomarker of several types of cancer^51,52^ and that BPA has the ability to modify SOD2 levels in different cell types^53,54^. Therefore, up-regulation of these proteins in 100 µM BPA-treated germ cells could be associated with abnormal cell growth, cellular damage, and cancer. Unlike the other proteins, PHB showed maximal expression at 0.01 µM BPA treatment and relatively higher expressions at other BPA doses than control. Heat shock proteins (HSPs) are generally responsible for structural formation of several cellular proteins and the prevention of protein misfolding. HSPE1 level increased at 100 µM BPA treatment, whereas HSPD1 and HSPA8 level decreased following the same treatment, indicating the possibility of the formation of abnormal decorating spindles and centrosomes during cell division, as reported in several studies, especially on 70 kDa HSP^55^. Furthermore, expression of the enzyme GLT1D1, which is responsible for the formation of different types of polysaccharides, was remarkably down-regulated by 100 µM BPA treatment. Additionally, as shown in Fig. 7B, almost all of the identified proteins are interrelated via regulatory networks, suggesting simultaneous up- or down-regulation on the expression of these proteins in germ cells due to BPA administration. As the proteins are interlinked, BPA induced alteration of the expression of oxidative stress (SOD2, PRDX4) and apoptosis (PPIA) related proteins can regulate HSPs and may affect fertility associated cell processes by triggering fertility related proteins such as PHB. Therefore, based on these findings, we conclude that BPA can cause effects on differentially expressed cellular proteins related to cell survival, growth and apoptotic pathways, including oxidative stress initiation, cellular immune response, and mitochondrial survivability (Fig. 7A). In other word, these findings indicate the possibility that BPA exposure may lead to the initiations of some latent diseases (shown in Fig. 7A) such as neoplasm and necrosis, together with delayed sex maturation (due to reduced expression of HSPA8) and infertility.

## Conclusion

To the best of our knowledge, this is the first comprehensive study on the effect of BPA exposure on the-proliferation, survivability, stemness properties, and cellular proteome of testicular germ cells, based on *in vitro* culture method. Low concentrations of BPA (0.01, 0.1, and 1 µM) did not produce significant or partial toxic effects in cultured germ cells, whereas 10 and 100 µM BPA doses resulted in apoptosis, altered stem cells functional abilities possibly triggering genetic abnormalities. This study also provides information about germ cell survivability and stemness properties under continuous BPA exposure. Finally, the up-regulation of proteins related to cancer, oxidative stress, and apoptosis in BPA-treated germ cell cultures suggests the possibility that BPA might be associated with various health risks and infertility. From this perspective, this study could be used as a starting point of examining the effects of other environmentally abundant EDCs on human health issues including reproduction.

## Methods

### Reagents

Reagent related information is described in Supplementary Methods section.

### Experimental Animals

Wild-type breeding stocks of male and female ICR (CD-1) mice (DBL, Korea) and C57BL/6-TG-EGFP (designated C57 GFP; Jackson Laboratory, Bar Harbor, Maine, USA) strain were used. All animals were cared and experimental protocols were approved according to the Animal Care and Use Committee of Chung-Ang University (IACUC Number: 2016-00009) and the *Guide for the Care and Use of Laboratory Animals* published by National Institutes of Health. The recipient C57BL/6 mice used for germ cell transplantation were obtained from Harlan Laboratories (Indianapolis, IN, USA). Mice were cared in epoxide cages and fed a diet containing minute amount of soy (has no soy related effects). Water was provided in BPA-free drinking bottles made of polysulfone to eliminate basal BPA exposure. Food and water were provided *ad libitum*.

### Magnetic activated cell sorting (MACS) and germ cell culture with BPA

MACS, which is generally used for isolation and culture of testis cells from mouse pup (6–8 days old) for enrichment of SSCs, was performed as previously described^56^. Germ cell culture was then set to enhance the number of cells stable enough for further experiment progressing. The detailed procedure is described in Supplementary Methods section.

BPA (239658, Sigma, St. Louis, MO, USA) was dissolved in 100% dimethyl sulfoxide (DMSO; D2650, Sigma, St. Louis, MO, USA), a 10-fold serial dilution series, ranging in concentration from 0.01 to 100 mM, was prepared in DMSO. 0.1% (v/v) from each of the BPA concentration was mixed with mSFM so that the final concentration reached to µM level. Thus, germ cell culture of CD-1 and C57 GFP mice were prepared with BPA-mixed medium and media were changed every 24 h. Control groups received medium containing 0.1% (v/v) DMSO. After 1 week culture with different BPA concentrations, harvested germ cells were counted by hemocytometer. Germ cell proliferation was measured by counting total number of harvested cells from culture and viability was assessed by calculating percentage of viable cells with trypan blue exclusion test^57^. Four replicates of each germ cell culture were used for data analysis.

### Analysis of germ cell apoptosis

To observe the effect of BPA on germ cell apoptosis, the percentage of apoptotic cells was examined immediately after harvesting cultured cells. The detailed procedure is described in Supplementary Methods section.

### Immunocytochemistry

SSC self-renewal related marker protein (PLZF), undifferentiated spermatogonia surface receptors (GFR α1 and RET), and differentiated spermatogonia marker protein (C-KIT) were selected to evaluate their expression in BPA-exposed cultured germ cells. Immunocytochemistry was performed following the procedure described preciously^58^. Briefly, BPA-treated cells were fixed with 4% paraformaldehyde (PFA) for 20 min and permeabilized with 0.1% Triton X-100 in PBS for 10 min at room temperature (RT). DPBS containing 5% (w/v) bovine serum albumin (BSA) was applied for 30 min at RT to avoid nonspecific antibody binding. Then germ cell fixed slides were separated into four groups, for four different marker proteins. Cells were incubated with monoclonal anti-mouse promyelocytic leukemia zinc finger (PLZF, 1:100; OP128, Calbiochem, USA), rabbit anti-human glial-derived neurotrophic factor family receptor alpha 1 (GFRα1, 1:100; sc-10716, Santacruz, Texas, USA), goat anti-mouse receptor tyrosine kinase (RET, 1:100; AF482, R&D, Minneapolis, MN, USA) and goat anti-mouse C-KIT (1:100; sc-1494, Santacruz, Texas, USA) primary antibodies at 4 °C overnight. Cells were washed in DPBS for 3 times, and incubated with Alexa fluor 568-conjugated Goat anti-mouse IgG (1:200; A-11004, Life technologies, USA), Alexa fluor 568-conjugated Goat anti-Rabbit IgG (1:200; A-11011, ThermoFisher, USA) or Alexa fluor 568-conjugated Donkey anti-Goat IgG (1:200: A-11057, Invitrogen, USA) secondary antibodies for 1 h at RT. The labeled cells were again washed three times with DPBS, mounted with VectaShield mounting media (Vector Laboratories, USA) containing DAPI, and analyzed by using a Nikon TE2000-U microscope with NIS Elements imaging software (Nikon, Chiyoda-ku, Tokyo, Japan). Germ cells from six individual cultures were used and the number of cells positive for PLZF, GFRα1, RET and C-KIT were counted form 5 random microscopic fields. Percentages of PLZF, GFRα1, RET and C-KIT expressing cells were calculated by dividing the labeled cell number by the total number of DAPI positive cells in the same microscopic field.

### Germ cell transplantation for evaluating SSC activity after BPA exposure

The transplantation was carried out as described by Brinster *et al*.^45,56^, to investigate the stemness state and changed characteristics of cultured germ cells with the presence of endocrine disruptor BPA. A similar mouse strain (C57BL/6) was used as recipient because the cultured germ cells were also derived from C57 GFP strain. The detailed procedure is described in Supplementary Methods section. Germ cells were injected in both testes and overall 8–10 recipient mice were used for control and each BPA group.

The number of colonies was calculated as the number of colonies per 10^5^ cells transplanted using the following equation: Colonies/10^5^ cells transplanted = Number of colonies × 10^5^/Number of cells transplanted. The total number of colonies was expressed as the number of colonies per total number of cells recovered after culture to indicate the potential effects of BPA on SSC proliferation, using the following equation: Colonies/Total number of cells cultured = Number of colonies × Total number of cells transplanted/Number of cells transplanted.

### Spermatocyte preparation and immunostaining

BPA-treated germ cell transplanted recipient mice were used to investigate the presence of meiotic abnormalities during spermatogenesis. Testes were collected from the recipient mice. Preparations of spermatocytes were processed according to the protocol of Peters *et al*.^59^ with a little modification where 1% PFA was applied as a thin layer over a clean slide rather than by dipping it into PFA. A drop of nuclear suspension was spread over the slides and then kept in a humid chamber for overnight, dried and washed with a solution of 0.4% Photo-Flo 200 (Kodak Professional, NY, USA). Finally slides were kept to air-dry.

Before immunofluorescence staining, sections were blocked with 5% BSA for 1 h at RT and incubated with primary antibodies overnight at 4 °C. After washing twice with PBS, subsequent secondary antibodies were applied for 1 h at RT. For observing spermatocyte status, mutl homolog 1 (MLH1, 1:60; PC56, Calbiochem, San Diego, CA, USA), RAD51 (1:60; sc-8349, Santacruz, Texas, USA) and synaptonemal complex protein 3 (SYCP3, 1:300; sc-74569, Santacruz, Texas, USA) were used along with DNA double strand break indicating rabbit polyclonal anti-γH2AX antibody (1:300; ab-11174, Abcam, MA, USA). Nuclear images were captured by epifluorescence microscope (Olympus U-TV1X-2, Tokyo, Japan). MLH1 foci were counted from almost all (at least 25–30) pachytene stage nuclei from 6–7 transplanted recipients and synaptonemal complex (SC) length were measured from the autosomes of the same nuclei using the measuring tool of the microscope. RAD51 foci counts were done from almost all zygotene stages per mice and SC length and RAD51 scores were averaged.

### Long-term culture with BPA and recovery of germ cells from BPA exposure

To test the effects of long-term BPA exposure, germ cells were treated with BPA concentrations ranging from 1 to 100 µM for 3 weeks. Subculture was performed every week on fresh STO feeder layer. Cells were seeded at a density of 0.2 × 10^6^ cells/well each week. If any BPA treated group showed cell number lower than 0.2 × 10^6^, all of the harvested cells from each wells were used for subculture.

In case of recovery test, germ cells were treated with 100 µM BPA for 1 week and then subcultured for the following 3 weeks without BPA treatment. Both the long-term cultured germ cells and recovered germ cells from BPA exposure were transplanted into recipient mice and 8–10 mice were used per group.

### Two dimensional gel electrophoresis (2-DE) analysis of BPA-induced proteome alterations in germ cells and pathway studio

A 2-DE-based comparative proteomic analysis was performed to investigate the changes of protein expressions on BPA effect and other procedures were followed according to described^20,60^. The detailed procedure is described in Supplementary Methods section.

### Statistics

Data were analyzed by one-way analysis of variance (ANOVA) using SPSS (version 12.0, IBM, USA) and Prism software (version 5.03; GraphPad, La Jolla, CA, USA). Analysis of variance was used to test for differences among treatment groups and each assay was run at least in triplicate. For identification of differences in the recovery test, student’s two-tailed t test was used. Significant differences between means were determined using Duncan and Tukey’s Honestly Significant Difference test where P < 0.05 was considered as significant.

### Data availability

The datasets generated and/or analyzed in the current study are available from the corresponding author on reasonable request.

## Electronic supplementary material

Supplementary Information
