# Supplementary material for: Bisphenol A Affects on the Functional Properties and Proteome of Testicular Germ Cells and Spermatogonial Stem Cells in vitro Culture Model
Source: Sci Rep. 2017 Sep 19;7:11858. doi: 10.1038/s41598-017-12195-9 (PMC5605497; doi:10.1038/s41598-017-12195-9)
Supplement: Supplementary file 1 — Supplementary Information [file 41598_2017_12195_MOESM1_ESM.pdf]

Supplementary Information for: Karmakar *et al*.

Bisphenol A Affects on the Functional Properties and Proteome of Testicular Germ Cells and Spermatogonial Stem Cells *in vitro* Culture Model

Polash Chandra Karmakar[1,+], Hyun-Gu Kang[1,+], Yong-Hee Kim[1], Sang-Eun Jung[1], Md Saidur Rahman[1], Hee-Seok Lee[2], Young-Hyun Kim[3,4], Myung-Geol Pang[1] and Buom-Yong Ryu[1],*

[1]Department of Animal Science & Technology, Chung-Ang University, Anseong, Gyeonggi-do 456-756, Republic of Korea.

[2]Food Safety Risk Assessment Division, National Institute of Food & Drug Safety Evaluation, Ministry of Food and Drug Safety, 187 Osongsaengmyeong2-ro, Osong-eup, Heungdeok-gu, Cheongju-si, Chungcheongbuk-do, Republic of Korea.

[3]National Primate Research Center, Korea Research Institute of Bioscience and Biotechnology(KRIBB), 30 Yeongudanji-ro, Ochang-eup, Cheongwon-gu, Cheongju-si, Chungcheongbuk-do, Republic of Korea.

[4]Department of Functional Genomics, KRIBB School of Bioscience, Korea University of Science and Technology(UST), 217 Gajeong-ro, Yuseong-gu, Daejeon, Republic of Korea.

Supplementary Methods

Reagents

Minimum essential medium α (MEMα; 12000-014, powder, Gibco, NY, USA) was used in the culture of testicular germ cells. SSC specified bovine serum albumin (BSA; A1470, Sigma, St. Louis, MO, USA) and growth factors such as glial cell line-derived neurotrophic factor (GDNF; 212-GD-50, R&D Systems, Minneapolis, MN, USA), GDNF family receptor α1 (GFRα1; 560-GR-100, R&D Systems, Minneapolis, MN, USA), and basic fibroblast growth factor 2 (bFGF2; 354060, BD Biosciences, San Jose, CA, USA) were used in culture. Dulbecco's modified Eagle's medium (DMEM; 12100-046, powder, Gibco, NY, USA) was used for culturing SIM mouse embryo-derived thioguanine- and ouabain-resistant (STO) feeder cells. Plastic items used for culture and reagent preparation were proposed with lowest potential background of BPA.

Magnetic activated cell sorting (MACS) and germ cell culture

Testis cells, including spermatogonia, were collected from the testes of CD-1 and C57 GFP mouse pups (6-8 days old). Pups were euthanized by CO_2, and testes were collected, decapsulated and washed with Dulbecco's phosphate buffered saline (DPBS; 14200166, Gibco, NY, USA). Seminiferous tubules were then incubated in a solution consisting 2:1 ratio of 0.25% trypsin-EDTA (25200056; Gibco, CA, USA) and 7 mg/mL DNase I (10104159001, Roche, Mannheim, Germany) dissolved in DPBS at 37°C for 5 min. 10% (v/v) fetal bovine serum (FBS; SH30919.03, Thermo Scientific, Utah, USA) was then added to inactivate the enzymatic reactions and a cell suspension was prepared. To remove debris, the suspension was filtered through a 40-µm pore size nylon mesh (BD Biosciences, San Jose, CA, USA). Cell viability was determined using the trypan blue exclusion test[1] (trypan blue solution; T8154, Sigma, St Louis, MO, USA), and testis cell preparations with a viability greater than 95% were used for subsequent steps. Cell suspension was then centrifuged at 600 x g for 7 min at 4°C and resuspended with suspension medium containing DMEM, 10% (v/v) FBS, 2 mM L-glutamine (25030, Gibco, SP, Brazil), 0.1 mM β-mercaptoethanol (M7522, Sigma, St Louis, MO, USA), and 100 U/mL penicillin with 100 µg/mL

streptomycin (15140122, Gibco, NY, USA). 30% Percoll solution was papered as 30% (v/v) Percoll (P1644, Sigma, St Louis, MO, USA), 1% (v/v) FBS, 0.5% (v/v) penicillin-streptomycin dissolved in deionized water (15230, Gibco, NY, USA). Cell suspension (2 mL, containing up to 10 x 10^6 cells) was overlaid on 2 mL of 30% Percoll solution and centrifuged at 600 x g for 10 min at 4°C to separate erythrocytes and cell debris from the suspension. The pellet was resuspended with suspension medium. Mouse anti-THY-1 positive (Thy-1^+) antibody with microbeads (Miltenyi Biotech, Auburn, CA, USA) was then used for separating undifferentiated germ cells by MACS as described previously[2]. After collection of germ cells from MACS, mouse serum free culture medium (mSFM, contains MEMα) was prepared and cell culture was prepared following the protocol described by Kubota *et al*.[3,4]. Briefly, cells were cultured with mSFM containing 10 ng/mL GDNF, 75 ng/mL GFRα1, and 1 ng/mL bFGF2 to enhance the number of germ cells. Culture plates (24-well) were prepared with a layer of mitotically inactivated STO feeder cells before germ cell seeding. Germ cells collected from CD-1 and C57 GFP mice were placed on this feeder layer as approximately 0.2 x 10^6 cells/well and incubated at 37°C in a 5% CO_2 atmosphere. Culture medium was changed in every 2-3 days. After 1 week, harvested cells were re-plated on new STO feeder layer. Two to three culture passages were continued in this way until the number of harvested cells was at least ~2-fold higher than the initial seeding number (0.2 x 10^6 cells/well), at which point the germ cells were presumed to be stable enough for further experiments and decided to culture in the presence of BPA.

Analysis of germ cell apoptosis

BPA-exposed cultured germ cells were examined for apoptosis following similar procedure described previously[5]. Cells were exposed to Annexin V-Phycoerythrin (PE) apoptosis kit (559763, BD Biosciences, San Diego, CA, USA) and the kit manufacturer's protocol was followed with slight modification. Briefly, germ cells were washed (centrifuged at 600 x g for 7 min at 4°C and resuspended) twice with ice cold DPBS immediately after harvested from culture. After this wash, cells were resuspended with 1X binding buffer (kit provided) as 2 x 10^5 cells/200 µL and 10 µL of Annexin V-PE

was added. Cells were incubated for 15 min in the dark at room temperature (RT) and the suspension volume was increased to 500 µL with 1X binding buffer. After that, propidium iodide (PI; P4170, Sigma, St. Louis, MO, USA) buffer was added to a final concentration of 5 µg/mL, and cells were placed on ice. Apoptotic cell populations were determined as a percentage of whole cell population using a FACS Aria flow cytometer (BD Biosciences CA, USA) with BD FACS Diva software. Germ cells collected from 6 individual cultures were used for this analysis.

Germ cell transplantation into recipient mice testes and analysis for donor cell derived colonies

Germ cell transplantation into recipient mice testes is the only way to precisely quantify SSCs in a germ cell population. Therefore, BPA exposed cultured germ cells were transplanted into recipient C57BL/6 mice. Six weeks old recipient mice were prepared for transplantation by injecting 45 mg/kg busulfan intraperitoneally (IP) to deplete endogenous spermatogenesis. On the day of transplantation, control and BPA-exposed cultured germ cells were harvested and prepared at a density of 1.0 $x10^6$ cells/mL with mSFM containing 10% (v/v) FBS and 10% DNase I (7 mg/mL). Before transplantation, recipient animals were anesthetized with 75 mg/kg ketamine and 0.5 mg/kg medetomidine. Lower abdominal hair was removed using a hair trimmer and the area was disinfected with iodine and 70% ethanol. A small surgical wound was then made and testis was driven out carefully from the abdomen. Next, donor germ cell suspension was labeled with 7% (v/v) trypan blue and was injected into the testes through efferent ducts as described peviously[6]. About 8-10 µL (~1.0 x 10^4 cells) of donor cell suspension were injected into each testis, filling ~80% of the surface seminiferous tubules.

Two and a half months after transplantation, the recipients were euthanized and the testes were collected and visualized under fluorescent microscope (Nikon AZ100, Tokyo, Japan). Testes were then decapsulated and the tubules were gently dispersed. The donor cell derived colonies were visualized separately and counted as previously described[7]; one colony (≥1 mm in length) was considered to have been produced from one SSC.

Two-dimensional gel electrophoresis (2-DE) analysis of BPA-induced proteome alterations in SSCs and pathway studio

Cell extracts from 1 week BPA-treated cultured germ cells were used to scrutinize differential protein expression by two-dimensional gel electrophoresis. Control group and 0.01, 0.1, 1, 10, and 100 μM BPA-treated groups were used. The fold difference in protein expression was expressed as the ratio of the intensity value of the spot derived from the BPA-treated group to the corresponding value for the control group. Three replications of cultured germ cells were used for this experiment.

Unless otherwise stated, all reagents were purchased from Sigma (St Louis, MO, USA). Protein extracts derived from cultured germ cells were incubated in a rehydration buffer containing 2 M thiourea, 7 M urea, 1% (w/v) octyl β-d-glucopyranoside, 24 μM PMSF, 4% (w/v) 3-[(3-cholamidopropyl) dimethylammonio]-1-propanesulfonate (USB, Cleveland, OH, USA), 1% (w/v) DTT, 0.05% (v/v) Triton X-100, 0.002% (w/v) bromophenol blue and 0.5% (v/v) IPG buffer at 4°C for 1 h. Thereafter, cellular proteins (250 μg) were solubilized and were placed in rehydration buffer (450 μL) in a rehydration tray with 18 cm-long NL Immobiline DryStrips (pH 3–11; Amersham, Piscataway, NJ, USA) at 4°C for 12 h. An IPGphor isoelectric focusing apparatus (Amersham) was applied for first-dimension electrophoresis. Then, the strips were focused for 1 h to each of the volts as 100 V, 200 V, 500 V, 1000 V and 1.5 h to 5000 V, 8000 V, and finally 1 h to 8000–90,000 V. The strips were then equilibrated with equilibration buffer A containing 6 M urea, 30% (v/v) glycerol, 75 mM Tris-HCl (pH 8.8), 2 % (w/v) SDS, 2 % (w/v) DTT and 0.002 % (w/v) bromophenol blue at RT for 15 min. Equilibration buffer B (equilibration buffer A with 2.5 % [w/v] iodoacetamide but without DTT) was used to equilibrate the strips for a second time at RT for 15 min. Second-dimension electrophoresis was carried out with 12.5 % (w/v) SDS-PAGE gels with the strips at 100 V for 1 h and 500 V until the bromophenol blue front began to migrate off the lower end of the gel. Following the manufacturer's instructions (Amersham), the gels were then silver-stained for image analysis and scanned using a high-resolution GS-800 calibrated scanner (Bio-Rad, Hercules, CA, USA). Detected protein spots were matched using PDQuest 8.0 software (Bio-Rad, Hercules, CA, USA) comparing the gels from BPA-treated germ cells and control. Finally, the densities of selected spots

were calculated and normalized as the ratio of the spot density in BPA-treated germ cells to the spot density in control.

In-gel protein identification by in-gel digestion, desalting and concentration technique was performed as described previously[8,9]. Mass spectrometry (MS) was used to generate peptide from in-gel digested proteins using tandem nano-electrospray ionization (ESI) on a MicroQ-TOF III mass spectrometer (Bruker Daltonics) at RT. The ESI-MS/MS data were analyzed using a peptide sequence system. MASCOT software (Matrix Science) was used to search for MS/MS ions. Peptide fragments attained from peptide peaks in ESI-MS by ESI-MS/MS were then identified using the Mascot search engine (Matrix Science). The results were limited to *Mus musculus*.

The Pathway Studio (version 9.0, Ariadne Genomics, MD, USA) classification system was used to predict the signaling pathways involved in the response to 0.01, 0.1, 1, 10, and 100 μM BPA treatment and to understand the biological relevance of the observed changes in protein expression. The identified proteins were classified into several categories according to their functions during biological processes. Proteins involved in oxidative stress response, cell proliferation, and apoptosis, as well as heat shock proteins, accounted for the majority of the detected proteins. Protein–protein interactions among the identified proteins were also predicted based on the STRING system (version 10.0, online).

Supplementary Figure Legend:

Figure S1. Representative silver nitrate stained image of 2-DE protein spots. Protein spots of spermatogonial stem cells cultured with different concentrations of BPA are represented as (A) control, (B) 0.01 μM BPA, (C) 0.1 μM BPA, (D) 1 μM BPA, (E) 10 μM BPA, and (F) 100 μM BPA.

Supplementary Figure S1

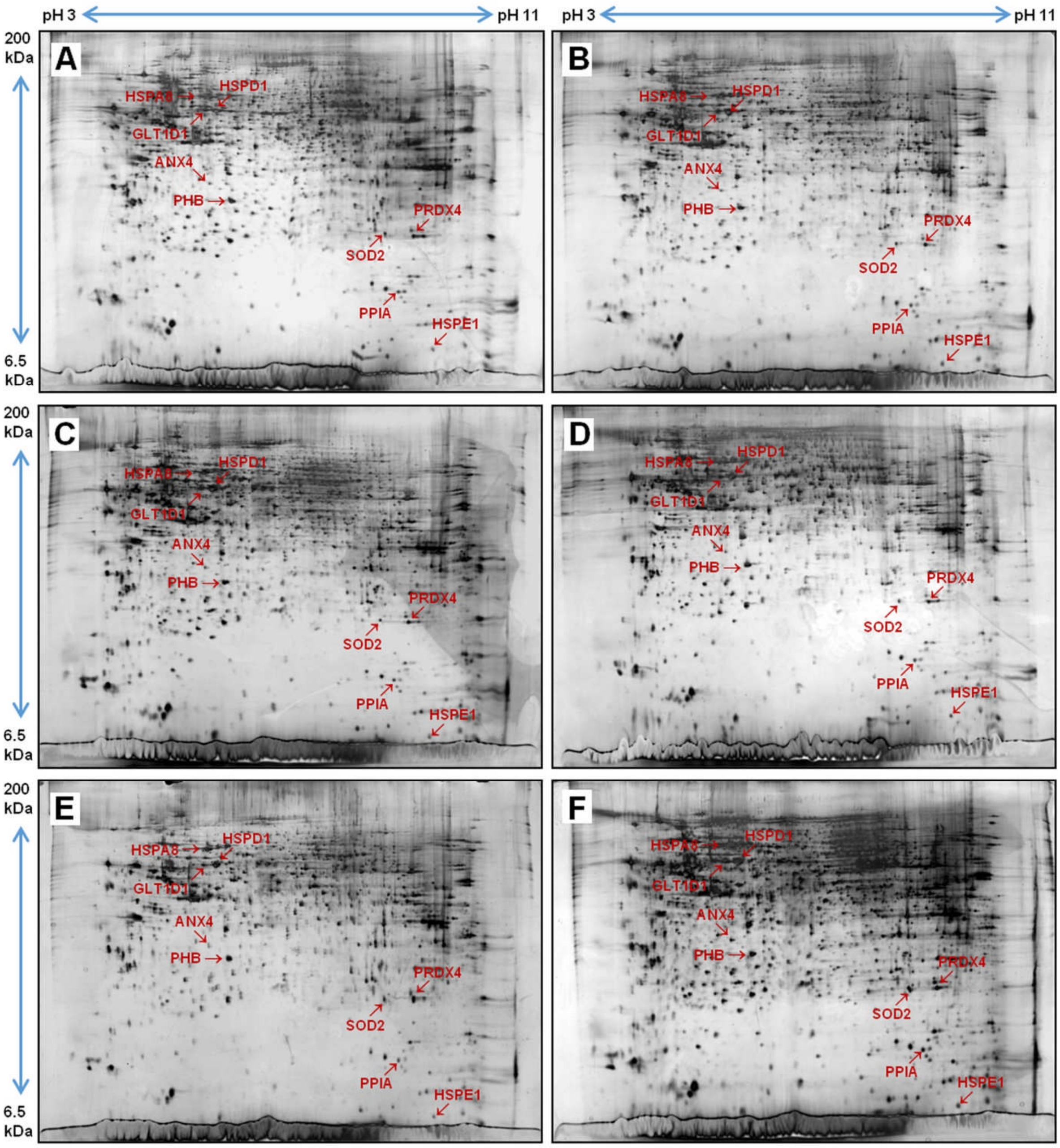

Supplementary Figure S1

Supplementary References:

1. Strober, W. Trypan blue exclusion test of cell viability. *Curr Protoc Immunol* **Appendix 3**, Appendix 3B, doi:10.1002/0471142735.ima03bs21 (2001).
2. Oatley, J. M. & Brinster, R. L. Spermatogonial stem cells. *Methods Enzymol* **419**, 259-282, doi:10.1016/S0076-6879(06)19011-4 (2006).
3. Kubota, H., Avarbock, M. R. & Brinster, R. L. Culture conditions and single growth factors affect fate determination of mouse spermatogonial stem cells. *Biol Reprod* **71**, 722-731, doi:10.1095/biolreprod.104.029207 (2004).
4. Kubota, H., Avarbock, M. R. & Brinster, R. L. Growth factors essential for self-renewal and expansion of mouse spermatogonial stem cells. *Proc Natl Acad Sci U S A* **101**, 16489-16494, doi:10.1073/pnas.0407063101 (2004).
5. Lee, Y. A. *et al.* Cryopreservation of mouse spermatogonial stem cells in dimethylsulfoxide and polyethylene glycol. *Biol Reprod* **89**, 109, doi:10.1095/biolreprod.113.111195 (2013).
6. Ogawa, T., Aréchaga, J.M., Avarbock, M.R. & Brinster, R.L. Transplantation of testis germinal cells into mouse seminiferous tubules. *Int J Dev Biol.* **41**(1), 111-22 (1997).
7. Nagano, M., Avarbock, M.R. & Brinster, R.L. Pattern and kinetics of mouse donor spermatogonial stem cell colonization in recipient testes. *Biol Reprod*. **60**(6), 1429-36 (1999).
8. Kwon, W. S. *et al.* A comprehensive proteomic approach to identifying capacitation related proteins in boar spermatozoa. *Bmc Genomics* **15**, 897, doi:10.1186/1471-2164-15-897 (2014).
9. Kwon, W. S. *et al* Discovery of Predictive Biomarkers for Litter Size in Boar Spermatozoa. *Molecular & Cellular Proteomics* **5**, 897, doi:10.1074/mcp.M114.045369 (2015).
